# LncRNA PVT1 regulates prostate cancer cell growth by inducing the methylation of miR‐146a

**DOI:** 10.1002/cam4.900

**Published:** 2016-10-28

**Authors:** Hong‐tao Liu, Lei Fang, Yu‐xia Cheng, Qing Sun

**Affiliations:** ^1^ Department of PathologyQian‐fo‐shan Hospital Affiliated to Shandong UniversityNo. 16766 Jingshi RoadJinanShandong250014China

**Keywords:** Growth, methylation, miR‐146a, prostate cancer, PVT1

## Abstract

Prostate cancer is the third most common causes of death from cancer in men. Our previous study demonstrated that lncRNA PVT1 was overexpressed and played an oncogenic role in the progression of prostate cancer. However, the molecular mechanism of modulating the prostate cancer tumorigenesis was still unknown. In this study, we aim to investigate the interaction between PVT1 and miR‐146a in prostate cancer and reveal the potential mechanism in prostate cancer carcinogenesis. The expression level of miR‐146a was assessed by quantitative RT‐PCR. The correlation analysis and methylation status analysis was made to confirm the interaction between PVT1 and miR‐146a. Biological function analysis was performed through gain‐of‐function and loss‐of‐function strategies. Our results showed that miR‐146a was downregulated and negatively correlated with PVT1 level in prostate cancer. PVT1 mediated miR‐146a expression by inducing the methylation of CpG Island in its promoter. miR‐146a overexpression eliminated the effects of PVT1 knockdown on prostate cancer cells. PVT1 regulated prostate cancer cell viability and apoptosis depending on miR‐146a. Our study suggested a regulatory relationship between lncRNA PVT1 and miR‐146a during the process of the prostate cancer tumorigenesis. PVT1 regulated prostate cancer cell viability and apoptosis depending on miR‐146a. It would contribute to the diagnosis, treatment and prognosis of prostate cancer.

## Introduction

Prostate cancer is the most common male reproductive tumor the third most common causes of death from cancer in men in the whole world 1, 2, 3, 4. Although the therapeutic options, such as radical prostatectomy and radiation, could successfully cure the majority of patients, approximately 30–40% of patients will relapse. It is the major cause impairing survival of the patients with prostate cancer 5, 6, 7. Understanding the pathogenesis of prostate cancer and identifying the target for the early detection and therapy of prostate cancer is urgently required.

Long‐noncoding RNA (lncRNA) plasmacytoma variant translocation 1 (PVT1), located at 8q24.21, is associated with the cell proliferation, apoptosis, lymph node invasion and metastasis, and tumor prognosis 8, 9, 10, 11, 12. A large number of studies have focused on its biological function in tumor progression. It was confirmed to be aberrant expression in gastric cancer, hepatocellular carcinoma, nonsmall cell lung cancer, ovarian, and breast cancer 8, 9, 10, 13, 14. Recent study found that PVT1 was upregulated in prostate cancer and identified PVT1 as the oncogene to increase the risk of prostate cancer 15, 16. In our previous study, we also demonstrated that PVT1 was overexpressed and played an oncogenic role in the progression of prostate cancer. However, the target gene of PVT1 and the molecular mechanism of modulating the prostate cancer tumorigenesis were still unknown.

miR‐146a was confirmed to be associated with the risk of various cancers, including gastric cancer, hepatocellular carcinoma, lung cancer, breast and ovarian cancer, bladder cancer, prostate cancer, etc 17, 18, 19, 20, 21, 22, 23, 24. Functional study showed that miR‐146a played paradoxical roles in various human cancer tissues. It exerted functions as an oncogene in hepatocellular carcinoma and cervical cancer, but as a tumor suppressor in pancreatic cancer and breast cancer 25, 26, 27, 28, 29. A study on glioblastoma reported that high expression of miR‐146a could inhibit tumor growth and migration of glioma stem‐like cells by downregulating Notch‐1 30. Overexpression of miR‐146a was confirmed to suppress the migration and invasion of gastric cancer cells 31. Transfection of mir‐146a in androgen‐independent PC3 cells was markedly reducing cell proliferation, invasion, and metastasis 32. In castration‐resistant prostate cancer, miR‐146a suppressed tumor growth and progression by targeting EGFR pathway. However, whether there was interaction relationship between miR‐146a and PVT1 in prostate cancer tumor progression was unclear.

In this study, we aim to explore the interaction between lncRNA PVT1 and miR‐146a in prostate cancer and reveal the potential mechanism in prostate cancer carcinogenesis. Our results suggested that PVT1 exhibited oncogenic activity through the negative regulation of miRNA‐146a by inducing the methylation in its promoter.

## Materials and Methods

### Patients

Prostate cancer samples and adjacent noncancerous tissues were obtained from Qian‐fo‐shan Hospital Affiliated to Shangdong University. The diagnosis of prostate cancer was performed according to World Health Organization criteria. Informed consent for the collection of samples was obtained from all patients and donors. This study protocol was approved by the Medical Ethics Committee of Qian‐fo‐shan Hospital Affiliated to Shangdong University.

### Cell culture

The human prostate cancer cell lines LNCaP, PC‐3 and DU145 were purchased from the American Type Culture Collection (ATCC). Cultures were maintained at 37°C in an atmosphere of 5% CO_2_.

### Cell viability and apoptosis assay

Cells were seeded into 96‐well plates at 2 × 10^3^ cells/well. Cell viability was detected using the MTT Cell Proliferation/Viability Assay kit (Sigma, Germany) according to the guidelines. Experiments were independently repeated three times.

Cells were seeded at a density of 1 × 10^6 ^cells/well in six‐well plates. Apoptosis was evaluated using an Annexin‐V‐Fluos and Propidium Iodide (PI) Apoptosis Detection Kit (Sigma, Germany) by fluorescence activated cell sorter (FACS) according to the manufacturer's protocol. Experiments were independently repeated three times.

### Quantitative RT‐PCR and MSP

Total mRNA was isolated using TRIzol Reagent (Takara, Japan) and reverse transcribed into cDNA using Prime Script RT reagent kit (Takara, Japan) according to the manufacturer's protocol. Real‐time PCR was performed by the SYBR Premix Ex TaqTM II kit (Takara, Japan) on a Stratagene MX3005P system (Agilent, Santa Clara, CA, USA). 5 *μ*L was then subjected to Methylation‐specific PCR (MSP) with Power SYBR Green PCR Master Mix (Applied Biosystems, Shanghai, China) according to the manufacturer's guidelines. GAPDH served as an internal standard. Experimental operation and reaction conditions referred to the description previously 33.

### Cell infection and transfection

PVT1 siRNA/negative control siRNA and PCDNA3‐PVT1/PCDNA3‐Ctrl were synthesized from Santa Cruz Biological (Santa Cruz, Paso Robles, CA, USA). miR‐146a target vector/empty vector and (LNA‐anti‐miR‐146a)/negative control inhibitor (LNA‐Ctrl) were purchased from Exiqon (Copenhagen, Denmark). Cells were grown on six‐well plates to confluency and, respectively, transfected using Lipofectamine 2000 (Invitrogen, Carlsbad, CA, USA). Cells were harvested 48 h post transfection for qRT‐PCR to determine the transfection efficiency.

### Statistical analysis

Data were presented as mean ± standard deviation (SD). Statistical analysis was performed using SPSS software version 16.0. Comparisons of continuous data were analyzed using the independent *t*‐test between the two groups, whereas categorical data were analyzed by the *χ* ^*2*^ test. *P *<* *0.05 was considered statistically significant.

## Results

### Expression level of miR‐146a is downregulated and negatively correlated with PVT1 level in prostate cancer

**Figure 1:**
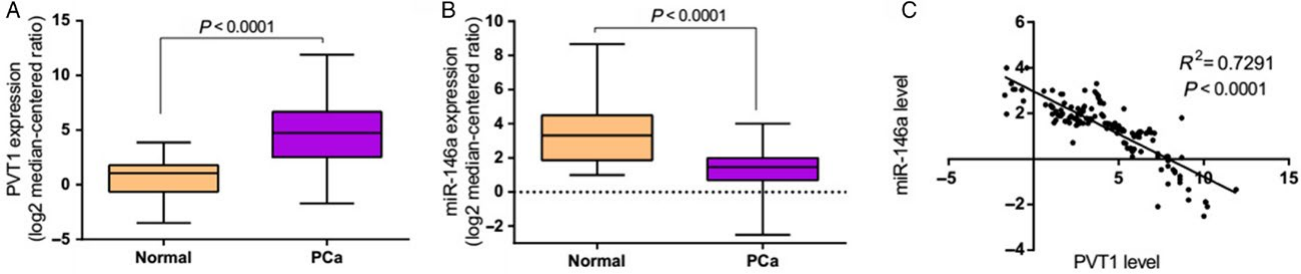
PVT1 was overexpressed in prostate cancer and negatively correlated with miR‐146a expression. (A) The expression level of PVT1 was upregulated in prostate cancer tissues. (B) The expression level of miR‐146a was downregulated in prostate cancer tissues. (C) Expression level of PVT1 was negatively correlated with miR‐146a. RNA was extracted from human prostate cancer tissue and adjacent normal tissues. The expression level of PVT1 and miR‐146a was analyzed by qRT‐PCR. GAPDH served as an internal standard. *n* = 152 in PCa group, *n* = 30 in normal group.

In our previous study, it has been found that PVT1 was overexpressed in prostate cancer and promoted prostate cancer growth in vivo and in vitro. Due to close association between miR‐146a and the risk of various cancers 17, 18, 19, 20, 21, 22, 23, 24, we speculated that miR‐146a may participate in the progress of prostate cancer. To explore whether miR‐146a involved in the tumorigenesis of prostate cancer, we firstly analyzed the expression pattern of miR‐146a in prostate cancer tissues. As shown in Figure 1, the mRNA level of miR‐146a was significantly downregulated in prostate cancer tissues (*P *<* *0.0001), whereas the PVT1 expression was obviously upregulated (*P *<* *0.0001). Linear regression analysis showed that the expression level of miR‐146a was negatively correlated with the PVT1 in prostate cancer (Fig. 1C, *R* ^*2*^ = 0.7291, *P *<* *0.0001).

### PVT1 regulates miR‐146a expression by inducing the methylation of CpG Island in its promoter

**Figure 2:**
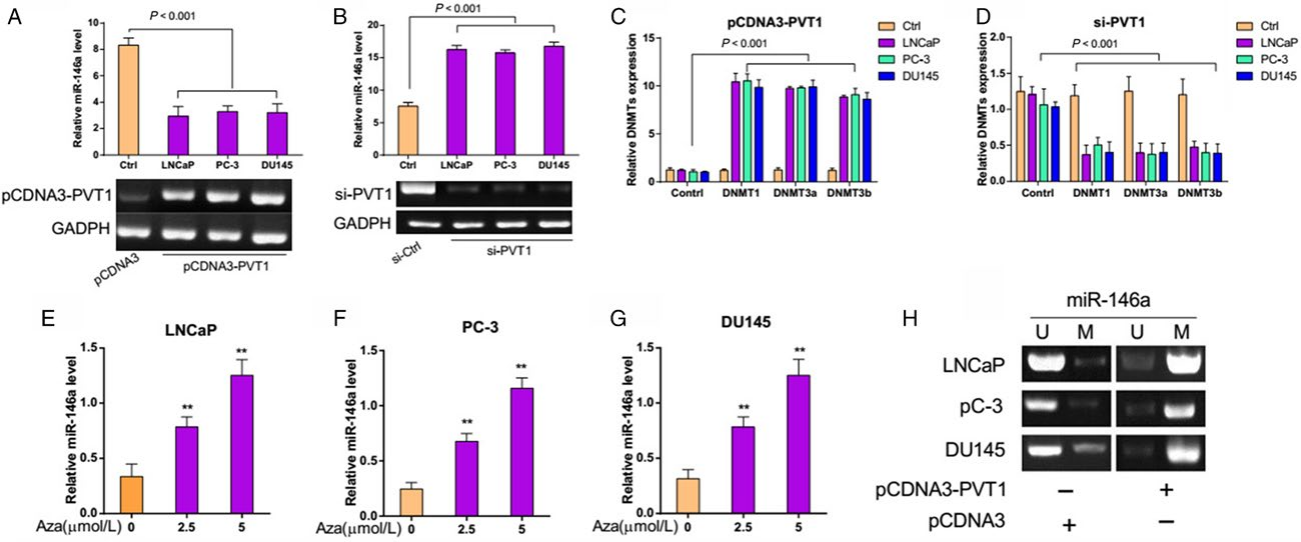
PVT1 regulated the expression of miR‐146a by inducing the methylation of CpG Island in its promoter. (A–B) miR‐146a expression level was down‐regulated with PVT1 overexpression and upregulated with PVT1 silencing. The expression level of miR‐146a was measured by qRT‐PCR in LNCaP, PC‐3, and DU145 cells transfected with either PCDNA3‐PVT1 or si‐PVT1. (C–D) DNMT1, DNMT3a, and DNMT3b expression levels were increased with PVT1 overexpression, and decreased with PVT1 silencing. The level of DNMT1, DNMT3a, and DNMT3b were analyzed using qRT‐PCR. (E–G) Methylation inhibitor 5‐azacytidine increased the miR‐146a expression. The expression level of miR‐146a was examined by qRT‐PCR in LNCaP, PC‐3, and DU145 cells. (H) PVT1 overexpression promoted the methylation of miR‐146a CpG islands. The methylation of miR‐146a CpG sites was analyzed by Methylation‐specific PCR (MSP) analysis in LNCaP, PC‐3, and DU145 cells. **P < 0.01

To further investigate the relationship between PVT1 and miR‐146a, we evaluated the expression of miR‐146a in three prostate cancer cell lines (LNCaP, PC‐3 and DU145) transfected with either PCDNA3‐PVT1 or si‐PVT1. Apparently the expression of PVT1 was increased in cells transfected with PCDNA3‐PVT1, but decreased in cells transfected with si‐PVT1 (Fig. S1). As shown in Figure 2A, the expression of miR‐146a was significantly inhibited in LNCaP, PC‐3 and DU145 cells when PVT1 was overexpressed (*P *<* *0.001). In contrast, PVT1 silencing markedly promoted miR‐146a expression in prostate cancer cells (Fig. 2B, *P* < 0.001). It implied that PVT1 regulated the expression of miR‐146a. To explore the mechanism of negative regulation of miR‐146a by PVT1, we analyzed the level of three active DNA methyltransferases (DNMT1, DNMT3a, and DNMT3b) in prostate cancer cell lines using qRT‐PCR when PVT1 was aberrantly expressed. It was found that the expression level of DNMT1, DNMT3a, and DNMT3b were obviously increased when PVT1 was overexpressed (Fig. 2C, *P* < 0.001). And the contrary result was observed when PVT1 was knocked‐down (Fig. 2D, *P* < 0.001). These results suggested that PVT1 might participate in the regulation of miR‐146a methylation. The methylation inhibitor, aza (5‐azacytidine), was used to demethylate the miR‐146a promoter. As shown in Figure 2E–G, aza increased the expression of miR‐146a in a concentration‐dependent manner in prostate cancer cell lines. In addition, MSP analysis provided evidences that PVT1 overexpression promoted the methylation of miR‐146a CpG islands (Fig. 2H). Taken together, these finding indicated that in prostate cancer, PVT1 regulated miR‐146a expression through inducing the methylation of CpG Island in its promoter.

### PVT1 regulates prostate cancer cell growth depending on miR‐146a

**Figure 3:**
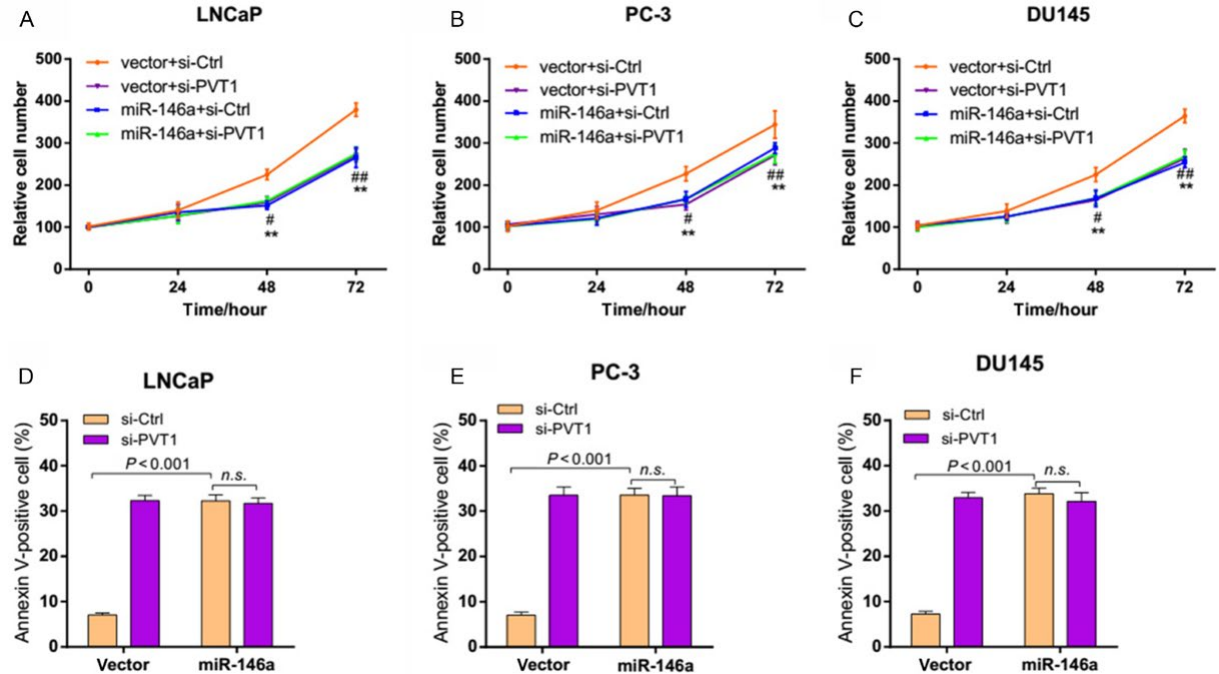
miR‐146a overexpression eliminated the effects of PVT1 knockdown on prostate cancer cells. (A–C) miR‐146a overexpression eliminated the effect of PVT1 knockdown on cell viability of prostate cancer cells. LNCaP, PC‐3, and DU145 cells were transfected with empty vector or miR‐146a target vector for 24 h and then infected with si‐PVT1. Relative cell numbers were evaluated using MTT analysis at the indicated time points. **P <* *0.01 indicates victory+si‐Ctrl versus vector+si‐PVT1, ^#^ *P *<* *0.05, ^##^ *P *<* *0.01 indicate vector+si‐Ctrl versus miR‐146a+si‐Ctrl. (D–F) miR‐146a overexpression blocked the effect of PVT1 knockdown on the apoptosis of prostate cancer cells. The percentage of apoptotic cells were analyzed with FACS. n.s., no significance; FACS, fluorescence activated cell sorter.

**Figure 4:**
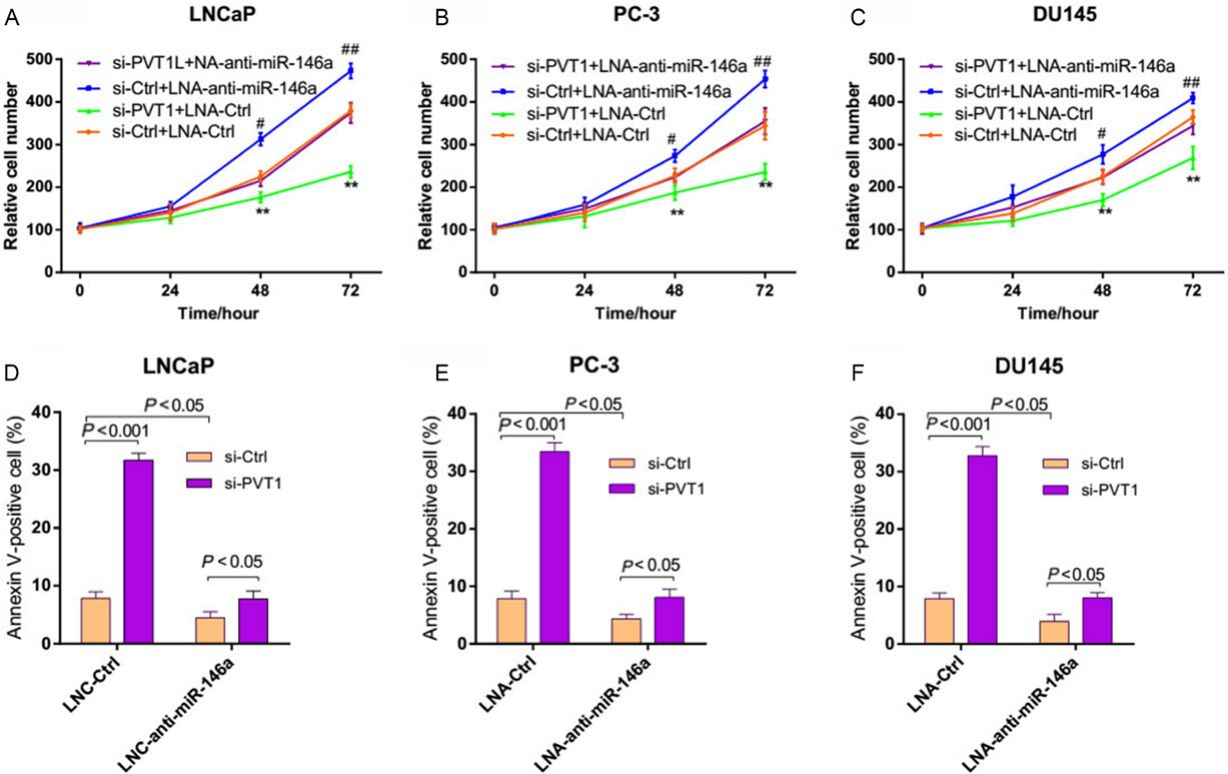
miR‐146a silencing counteracted the effects of PVT1 knockdown on prostate cancer cells. (A–C) miR‐146a silencing counteracted the effect of PVT1 knockdown on cell viability of prostate cancer cells. LNCaP, PC‐3, and DU145 cells were infected with LNA‐Ctrl or LNA‐anti‐miR‐146a for 24 h and then infected with si‐PVT1. Relative cell numbers were evaluated using MTT analysis at the indicated time points. **P <* *0.01 indicates si‐Ctrl+LNA‐Ctrl versus si‐PVT1 + LNA‐Ctrl, ^#^ *P *<* *0.05, ^##^ *P *<* *0.01 indicates si‐Ctrl+LNA‐Ctrl versus si‐Ctrl+ LNA‐anti‐miR‐146a. (D–F) miR‐146a silencing counteracted the effect of PVT1 knockdown on the apoptosis of prostate cancer cells. The percentage of apoptotic cells were analyzed with FACS. FACS, fluorescence‐activated cell sorter.

The significant aberrant expression of miR‐146a in prostate cancer tissues and negative regulation relationship with PVT1 suggested possible biological significance in tumorigensis. To investigate the biological roles of PVT1 and miR‐146a in prostate cancer, we applied gain‐of‐function and loss‐of‐function strategies. Knockdown of miR‐146a was evidenced by marked decrease in miR‐146a expression while significantly increased miR‐146a expression was observed in cells transfected with miR‐146a target vector (Fig. S1). It was demonstrated that PVT1 knockdown caused a significant reduction in cell viability and a promotion in cell apoptosis (Fig. 3, *P* < 0.001). Similarly, miR‐146a overexpression significantly inhibited cell survival and accelerated cell apoptosis in prostate cancer (Fig. 3, *P* < 0.001). But miR‐146a overexpression eliminated the effect of PVT1 knockdown on cell proliferation and apoptosis in prostate cancer cells (Fig. 3). Furthermore, as shown in Figure 4, miR‐146a silencing significantly promoted the cell viability, suppressed cell apoptosis. And the antitumor effect of PVT1 knockdown was counteracted when miR‐146a was silenced in prostate cancer cells. These results suggested that PVT1 regulated prostate cancer cell viability and apoptosis depending on miR‐146a.

## Discussion

LncRNA PVT1, a powerful predictive factor of tumor progression and patient survival in various cancers, has been demonstrated to play important roles in various biological processes, such as proliferation, apoptosis, mobility, and invasion 8, 9, 10, 11, 12. In our previous study, we confirmed that PVT1 predicted patient prognosis and regulated tumor growth in prostate cancer. However, the underlying molecular mechanism and related target genes were still unclear. In this study, we presented evidences that PVT1 exhibited oncogenic activity through the negative regulation of miRNA‐146a. PVT1 mediated the miR‐146a expression by inducing the methylation in its promoter.

miR‐146a was found to be associated with the risk of various cancers, including gastric cancer, hepatocellular carcinoma, lung cancer, breast and ovarian cancer, bladder cancer, prostate cancer, etc 17, 18, 19, 20, 21, 22, 23, 24. Some researchers have focused on its biological role and association with clinical response. It was found that miR‐146a decreased the sensitivity of HCC cells to the cytotoxic effects of IFN‐α through the suppression of apoptosis 25. Overexpression of miR‐146a was confirmed to suppress the migration and invasion of gastric cancer cells 31. Deregulation expression of miR‐146a affected EGFR signaling in pancreatic cancer model 34. In this study, it was found that miR‐146a was significantly low expression in prostate cancer tissues, suggesting possible biological significance in tumorigensis of prostate cancer. In addition, the expression level of miR‐146a was negatively correlated with the PVT1 expression. It implied that there may be a certain relationship between PVT1 and miR‐146a in prostate cancer.

Further research indicated that PVT1 regulated miR‐146a expression through the methylation in miR‐146a promoter. PVT1 knockdown markedly promoted miR‐146a expression, whereas the expression of miR‐146a was significantly inhibited in prostate cancer cells transfected with PCDNA3‐PVT1. In addition, PVT1 overexpression obviously increased the activity of DNA methyltransferases (DNMT1, DNMT3a, and DNMT3b) in prostate cancer cells. MSP analysis also provided evidences that PVT1 overexpression promoted the methylation of miR‐146a CpG islands. DNA methylation played an important role in regulating the expression of tumor‐related genes 35. Abnormality of DNA methylation was a crucial cause for tumorigensis 36. In this study, the methylation inhibitor, Aza, was used to demethylate the miR‐146a promoter. It was showed that methylation inhibitor increased the expression of miR‐146a in prostate cancer cell lines. PVT1 suppressed the miR‐146a expression through promoting the methylation level. There's little research involved in the relationship between PVT1 and miRNA except for some PVT1‐encoded miRNAs, such as miR‐1204, miR‐1205, miR‐1206, miR‐1207‐5p, miR‐1207‐3p, and miR‐1208 37. It was reported that miR‐1207‐5p, as a PVT1‐derived microRNA, was abundantly expressed in kidney cells and increased the expression of TGF‐β1, PAI‐1, and FN1 in a PVT1‐independent manner 38. Our study first reported the regulatory relationship between PVT1 and miR‐146a in prostate cancer tumorigenesis. However, it was proved not to be a direct interaction in our following study. It might be a complex process involving multiple factors and worth further study.

Previous studies have showed that PVT1 was overexpressed in many kinds of human cancers and associated with the cell proliferation, apoptosis, lymph node invasion, metastasis, and tumor prognosis 8, 9, 10, 11, 12, 13, 14. Recent research indicated that upregulation of PVT1 inhibited apoptosis in gastric cancer cell lines, increased the expression of MDR1, MRP, mTOR, and HIF‐1α, promoted the development of multidrug resistance 39. PVT1 expression was found to be correlated with hormone sensitivity and androgen receptor status of prostate cancer cell lines in vitro 15. In this study, we investigated the biological function of miR‐146a in prostate cancer. It was demonstrated that miR‐146a silencing obviously increased cell viability and suppressed cell apoptosis. In contrast, miR‐146a overexpression significantly inhibited cell survival and accelerated cell apoptosis in prostate cancer. Furthermore, miR‐146a overexpression eliminated the inhibition effect of PVT1 knockdown on cell proliferation and apoptosis in prostate cancer cells. Due to the negative regulation relationship, the antitumor effect of PVT1 knockdown was counteracted when miR‐146a was silenced in prostate cancer cells. These results suggested that PVT1 regulated prostate cancer cell viability and apoptosis depending on miR‐146a. It provided additional evidence to the reciprocal repression between PVT1 and miR‐146a in a functional aspect.

Our study suggested a regulatory relationship between lncRNA PVT1 and miR‐146a in prostate cancer tumorigenesis. A better understanding of the molecular mechanism of modulating the prostate cancer tumorigenesis would contribute to the diagnosis, treatment, and prognosis of prostate cancer.

## Conflict of Interest

All the authors declare that they have no conflict of interest.

## Supporting information


**Figure S1.** Relative expression level of PVT1 and miR‐146a. (A–C) Relative expression level of PVT1 in LNCaP, PC‐3 and DU145 cells when PVT1 was overexpressed or knocked‐down. (D–F) Relative expression level of miR‐146a in LNCaP, PC‐3, and DU145 cells when miR‐146a was overexpressed or silenced. **P <* *0.01 versus Ctrl.

## References

[1] Siegel, R. , J. Ma , Z. Zou , and A. Jemal . 2014 Cancer statistics, 2014. CA Cancer J. Clin. 64:9–29. 24399786 10.3322/caac.21208

[2] Bilusic, M. , C. Heery , and R. A. Madan . 2011 Immunotherapy in prostate cancer: Emerging strategies against a formidable foe. Vaccine 29:6485–6497. 21741424 10.1016/j.vaccine.2011.06.088 PMC3605720

[3] Siegel, R. , C. DeSantis , K. Virgo , K. Stein , A. Mariotto , T. Smith , et al. 2012 Cancer treatment and survivorship statistics. CA Cancer J. Clin. 62:220–241. 22700443 10.3322/caac.21149

[4] Xue, G. , Z. Ren , Y. Chen , J. Zhu , Y. Du , D. Pan , et al. 2015 A feedback regulation between mir‐145 and DNA methyltransferase 3b in prostate cancer cell and their responses to irradiation. Cancer Lett. 361:121–127. 25749421 10.1016/j.canlet.2015.02.046

[5] Roehl, K. A. , M. Han , C. G. Ramos , J. A. Antenor , and W. J. Catalona 2004 Cancer progression and survival rates following anatomical radical retropubic prostatectomy in 3,478 consecutive patients: Long‐term results. J. Urol. 172:910–914. 15310996 10.1097/01.ju.0000134888.22332.bb

[6] Thompson, I. , J. B. Thrasher , G. Aus , A. L. Burnett , E. D. Canby‐Hagino , M. S. Cookson , et al. 2007 Guideline for the management of clinically localized prostate cancer: 2007 update. J. Urol. 177:2106–2131. 17509297 10.1016/j.juro.2007.03.003

[7] Boorjian, S. A. , J. A. Eastham , M. Graefen , B. Guillonneau , R. J. Karnes , J. W. Moul , et al. 2012 A critical analysis of the long‐term impact of radical prostatectomy on cancer control and function outcomes. Eur. Urol. 61:664–675. 22169079 10.1016/j.eururo.2011.11.053

[8] Zhang, Z. , Z. Zhu , B. Zhang , W. Li , X. Li , X. Wu , et al. 2014 Frequent mutation of rs13281615 and its association with pvt1 expression and cell proliferation in breast cancer. J. Genet. Genomics 41:187–195. 24780616 10.1016/j.jgg.2014.03.006

[9] Y. R. Yang , S. Z. Zang , C. L. Zhong , Y. X. Li , S. S. Zhao , and X. J. Feng . 2014 Increased expression of the lncrna pvt1 promotes tumorigenesis in non‐small cell lung cancer. Int. J. Clin. Exp. Pathol. 7:6929–6935. 25400777 PMC4230094

[10] Ding, C. , Z. Yang , Z. Lv , C. Du , H. Xiao , C. Peng , et al. 2015 Long non‐coding rna pvt1 is associated with tumor progression and predicts recurrence in hepatocellular carcinoma patients. Oncol. Lett. 9:955–963. 25624916 10.3892/ol.2014.2730 PMC4301564

[11] Ding, J. , D. Li , M. Gong , J. Wang , X. Huang , T. Wu , et al. 2014 Expression and clinical significance of the long non‐coding rna pvt1 in human gastric cancer. OncoTargets Ther. 7:1625–1630. 10.2147/OTT.S68854 PMC4172193 25258543

[12] Takahashi, Y. , G. Sawada , J. Kurashige , R. Uchi , T. Matsumura , H. Ueo , et al. 2014 Amplification of pvt‐1 is involved in poor prognosis via apoptosis inhibition in colorectal cancers. Br. J. Cancer 110:164–171. 24196785 10.1038/bjc.2013.698 PMC3887297

[13] Fang, X. Y. , H. F. Pan , R. X. Leng , and D. Q. Ye 2015 Long noncoding rnas: Novel insights into gastric cancer. Cancer Lett. 356:357–366. 25444905 10.1016/j.canlet.2014.11.005

[14] Guan, Y. , W. L. Kuo , J. L. Stilwell , H. Takano , A. V. Lapuk , J. Fridlyand , et al. 2007 Amplification of pvt1 contributes to the pathophysiology of ovarian and breast cancer. Clin. Cancer Res. 13:5745–5755. 17908964 10.1158/1078-0432.CCR-06-2882

[15] Soubra, A. , B. Konety , and A. Bagchi . 2015 Mp61‐06 increased pvt1 expression correlates with advanced stage and hormone resistance of prostate cancer. J. Urol. 193:e748–e749.

[16] Meyer, K. B. , A. T. Maia , M. O'Reilly , M. Ghoussaini , R. Prathalingam , P. Porter‐Gill , et al. 2011 A functional variant at a prostate cancer predisposition locus at 8q24 is associated with pvt1 expression. PLoS Genet. 7:e1002165. 21814516 10.1371/journal.pgen.1002165 PMC3140991

[17] Fu, B. , P. Song , M. Lu , B. Wang , and Q. Zhao . 2014 The association between mir‐146a gene rs2910164 polymorphism and gastric cancer risk: A meta‐analysis. Biomed. Pharmacother. 68:923–928. 25455160 10.1016/j.biopha.2014.10.002

[18] Xu, T. , Y. Zhu , Q. K. Wei , Y. Yuan , F. Zhou , Y. Y. Ge , et al. 2008 A functional polymorphism in the mir‐146a gene is associated with the risk for hepatocellular carcinoma. Carcinogenesis 29:2126–2131. 18711148 10.1093/carcin/bgn195

[19] Jeon, H. S. , Y. H. Lee , S. Y. Lee , J. A. Jang , Y. Y. Choi , S. S. Yoo , et al. 2014 A common polymorphism in pre‐microrna‐146a is associated with lung cancer risk in a korean population. Gene 534:66–71. 24144839 10.1016/j.gene.2013.10.014

[20] Pastrello, C. , J. Polesel , L. Della Puppa , A. Viel , and R. Maestro . 2010 Association between hsa‐mir‐146a genotype and tumor age‐of‐onset in brca1/brca2‐negative familial breast and ovarian cancer patients. Carcinogenesis 31:2124–2126. 20810544 10.1093/carcin/bgq184

[21] Mittal, R. D. , R. Gangwar , G. P. George , T. Mittal , and R. Kapoor . 2011 Investigative role of pre‐micrornas in bladder cancer patients: A case–control study in north india. DNA Cell Biol. 30:401–406. 21345130 10.1089/dna.2010.1159

[22] Xu, B. , N. H. Feng , P. C. Li , J. Tao , D. Wu , Z. D. Zhang , et al. 2010 A functional polymorphism in pre‐mir‐146a gene is associated with prostate cancer risk and mature mir‐146a expression in vivo. Prostate 70:467–472. 19902466 10.1002/pros.21080

[23] George, G. P. , R. Gangwar , R. K. Mandal , S. N. Sankhwar , and R. D. Mittal . 2011 Genetic variation in microrna genes and prostate cancer risk in north indian population. Mol. Biol. Rep. 38:1609–1615. 20842445 10.1007/s11033-010-0270-4

[24] Nikolic´, Z. Z. , D. L. S. Pavic´evic´ , V. D. Vukotic´ , S. M. Tomovic´ , S. J. Cerovic´ , N. Filipovic´ , et al. 2014 Association between genetic variant in hsa‐mir‐146a gene and prostate cancer progression: Evidence from serbian population. Cancer Causes Control 25:1571–1575. 25084752 10.1007/s10552-014-0452-9

[25] Tomokuni, A. , H. Eguchi , Y. Tomimaru , et al. 2011 Mir‐146a suppresses the sensitivity to interferon‐*α* in hepatocellular carcinoma cells. Biochem. Biophys. Res. Commun. 414:675–680. 21982769 10.1016/j.bbrc.2011.09.124

[26] Wang, X. , S. Tang , S. Y. Le , R. Lu , J. S. Rader , C. Meyers , and Z. M. Zheng . 2008 Aberrant expression of oncogenic and tumor‐suppressive micrornas in cervical cancer is required for cancer cell growth. PLoS ONE 3:e2557. 18596939 10.1371/journal.pone.0002557 PMC2438475

[27] Bhaumik, D. , G. Scott , S. Schokrpur , C. K. Patil , J. Campisi , and C. C. Benz . 2008 Expression of microrna‐146 suppresses nf‐κb activity with reduction of metastatic potential in breast cancer cells. Oncogene 27:5643–5647. 18504431 10.1038/onc.2008.171 PMC2811234

[28] Hurst, D. R. , M. D. Edmonds , G. K. Scott , C. C. Benz , K. S. Vaidya , and D. R. Welch . 2009 Breast cancer metastasis suppressor 1 up‐regulates mir‐146, which suppresses breast cancer metastasis. Cancer Res. 69:1279–1283. 19190326 10.1158/0008-5472.CAN-08-3559 PMC2754225

[29] Li, Y. , T. G. VandenBoom , Z. Wang , D. Kong , S. Ali , P. A. Philip , et al. 2010 Mir‐146a suppresses invasion of pancreatic cancer cells. Cancer Res. 70:1486–1495. 20124483 10.1158/0008-5472.CAN-09-2792 PMC2978025

[30] Mei, J. , R. Bachoo , and C. L. Zhang . 2011 Microrna‐146a inhibits glioma development by targeting notch1. Mol. Cell. Biol. 31:3584–3592. 21730286 10.1128/MCB.05821-11 PMC3165557

[31] Yao, Q. , Z. Cao , C. Tu , Y. Zhao , H. Liu , and S. Zhang . 2013 Microrna‐146a acts as a metastasis suppressor in gastric cancer by targeting wasf2. Cancer Lett. 335:219–224. 23435376 10.1016/j.canlet.2013.02.031

[32] Lin, S. L. , A. Chiang , D. Chang , and S. Y. Ying . 2008 Loss of mir‐146a function in hormone‐refractory prostate cancer. RNA 14:417–424. 18174313 10.1261/rna.874808 PMC2248249

[33] Hanoun, N. , Y. Delpu , A. A. Suriawinata , B. Bournet , C. Bureau , J. Selves , et al. 2010 The silencing of microrna 148a production by DNA hypermethylation is an early event in pancreatic carcinogenesis. Clin. Chem. 56:1107–1118. 20431052 10.1373/clinchem.2010.144709

[34] Ali, S. , A. Ahmad , A. Aboukameel , A. Ahmed , B. Bao , S. Banerjee , et al. 2014 Deregulation of mir‐146a expression in a mouse model of pancreatic cancer affecting egfr signaling. Cancer Lett. 351:134–142. 24839931 10.1016/j.canlet.2014.05.013 PMC4115205

[35] Lyko, F. , and R. Brown . 2005 DNA methyltransferase inhibitors and the development of epigenetic cancer therapies. J. Natl Cancer Inst. 97:1498–1506. 16234563 10.1093/jnci/dji311

[36] Hu, C. , and J. Zhou . 2009 Abnormality of DNA methylation modification and lung cancer. International Journal of Pathology and Clinical Medicine 5:398–402.

[37] Beck‐Engeser, G. B. , A. M. Lum , K. Huppi , N. J. Caplen , B. B. Wang , and M. Wabl . 2008 Pvt1‐encoded micrornas in oncogenesis. Retrovirology 5:4. 18194563 10.1186/1742-4690-5-4 PMC2257975

[38] Alvarez, M. L. , M. Khosroheidari , E. Eddy , and J. Kiefer . 2013 Role of microrna 1207‐5p and its host gene, the long non‐coding rna pvt1, as mediators of extracellular matrix accumulation in the kidney: Implications for diabetic nephropathy. PLoS ONE 8:e77468. 24204837 10.1371/journal.pone.0077468 PMC3808414

[39] Zhang, X. W. , P. Bu , L. Liu , X. Z. Zhang , and J. Li . 2015 Overexpression of long non‐coding rna pvt1 in gastric cancer cells promotes the development of multidrug resistance. Biochem. Biophys. Res. Commun. 462:227–232. 25956062 10.1016/j.bbrc.2015.04.121

