# Supplementary figures and images for: LncRNA PVT1 regulates prostate cancer cell growth by inducing the methylation of miR‐146a
Source: Cancer Med. 2016 Oct 28;5(12):3512–9. doi: 10.1002/cam4.900 (PMC5224852; doi:10.1002/cam4.900)

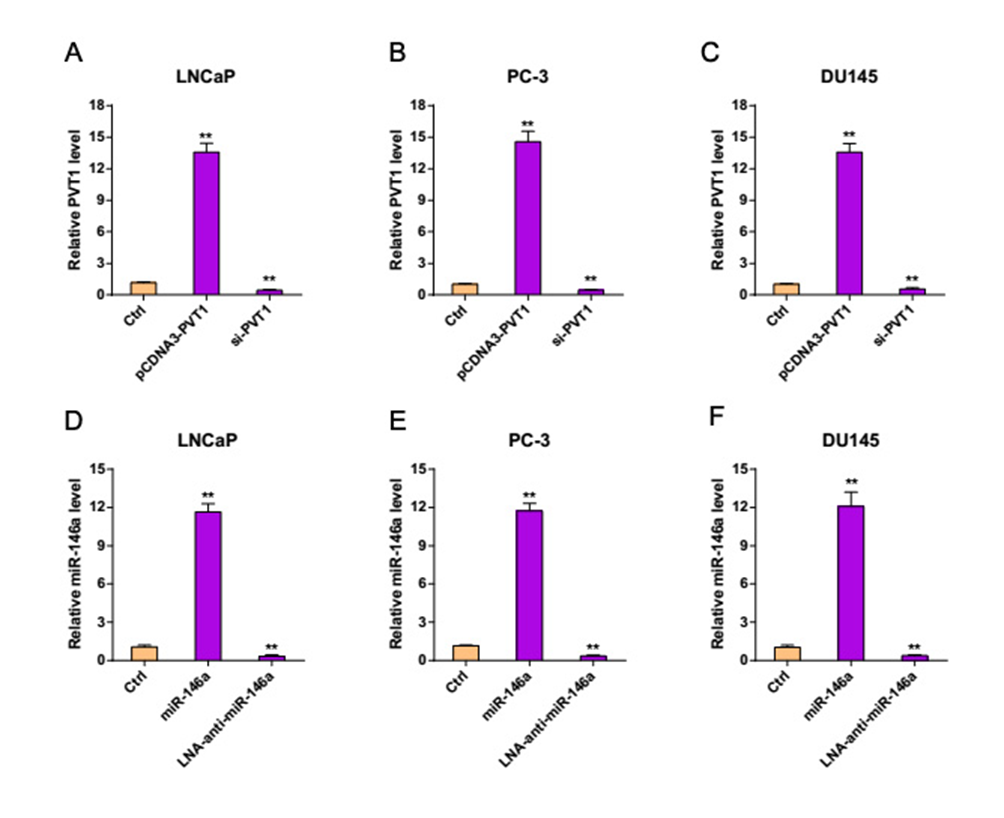

Supplement: Supplementary file 1 — Figure S1. Relative expression level of PVT1 and miR‐146a. (A–C) Relative expression level of PVT1 in LNCaP, PC‐3 and DU145 cells when PVT1 was overexpressed or knocked‐down. (D–F) Relative expression level of miR‐146a in LNCaP, PC‐3, and DU145 cells when miR‐146a was overexpressed or silenced. **P < 0.01 versus Ctrl. [file CAM4-5-3512-s001.tif]
